# Effects of lifestyle interventions on glucose regulation and diabetes risk in adults with impaired glucose tolerance or prediabetes: a meta-analysis

**DOI:** 10.20945/2359-3997000000441

**Published:** 2022-03-16

**Authors:** Qiang Jiang, Jian-Ting Li, Pei Sun, Lu-Lu Wang, Li-Zhi Sun, Shu-Guang Pang

**Affiliations:** 1 Shandong First Medical University Jinan Central Hospital Jinan Shandong China Jinan Central Hospital Affiliated to Shandong First Medical University, Jinan, Shandong, China

**Keywords:** Diabetes, adults, glucose tolerance, lifestyle interventions, risk

## Abstract

The prevalence of diabetes mellitus is increasing and is related to sedentary lifestyles and obesity. Many studies were published on the effect of lifestyle interventions on glucose regulation and delay the onset of diabetes in adults with impaired glucose tolerance (IGT) or prediabetes. This study aimed to investigate the role of lifestyle interventions in individuals with IGT or prediabetes using a meta-analytic approach. PubMed, Embase, and the Cochrane Central Register of Controlled Trials databases were searched from their inception up to January 2020 to select eligible randomized controlled trials (RCTs). The weighted mean difference (WMD; for fasting plasma glucose (FPG) and 2-hour plasma glucose (2hPPG)) or relative risk (RR; for the risk of diabetes) with 95% confidence interval (CI) were calculated for pooled effect estimates using the random-effects model. Thirteen RCTs involving 3376 individuals with IGT or prediabetes were selected for this meta-analysis. The results showed that lifestyle interventions were associated with lower FPG (WMD: -0.14; 95% CI: -0.24 to -0.05 mmol/L; p=0.004) and 2hPPG (WMD: -0.66; 95% CI: -1.12 to -0.20 mmol/L; p=0.005) in adults with IGT or prediabetes. Moreover, the risk of diabetes was significantly reduced in individuals who received lifestyle interventions (RR: 0.75; 95% CI: 0.60-0.95; p=0.015). Lifestyle interventions could help improve glucose dysregulation and prevent the progression of diabetes in adults with IGT or prediabetes. Further large-scale RCTs should be conducted to assess the effects of long-term lifestyle interventions on diabetic complications in adults with IGT or prediabetes.

## INTRODUCTION

The prevalence of diabetes mellitus (DM) is expected to rise from 463 million in 2019 to an estimated 578 and 700 million by 2030 and 2045, respectively, according to the International Diabetes Federation ( 1 ). The risk of mortality is significantly increased, and the life lost ranges from 12-14 years for adults with type 2 DM (T2DM), which are associated with an excess risk of cardiovascular disease, renal disease, and infection ( 2 ). Impaired glucose tolerance (IGT) or impaired fasting glucose (IFG) is an intermediate state of glucose dysregulation between normal glucose homeostasis and T2DM ( 3 ). Studies have found that nearly 70% of subjects with IGT or IFG could develop T2DM, and 20-30% of patients develop T2DM within 5-10 years ( 4 , 5 ).

Obesity and physical inactivity can increase the risk of T2DM, especially in individuals with IGT ( 6 ). Moreover, structured lifestyle interventions, including diet, behavior, and physical activity, were associated with modest weight reduction and could prevent the risk of T2DM ( 7 – 10 ). A meta-analysis of 71 studies conducted by Zhang and cols. found that lifestyle interventions significantly improved the fasting plasma glucose (FPG), HbA1c, fasting insulin, homeostasis model assessment-estimated insulin resistance, and body weight in healthy adults ( 11 ). Nevertheless, many studies were published on the effect of lifestyle interventions on glucose regulation and delay the onset of diabetes in adults with IGT or prediabetes ( 12 – 14 ). Gillies and cols. ( 13 ) examined pharmacological and lifestyle intervention randomized controlled trials (RCTs) in the prevention of T2DM. Balk and cols. ( 12 ) performed a meta-analysis of single-arm or observational studies of lifestyle changes on the risk of T2DM. Gong and cols. ( 14 ) examined the effect of lifestyle changes on glucose metabolism in IGT but did not examine the risk of diabetes. Furthermore, many studies were published since these previous meta-analyses were performed.

Therefore, we conducted this meta-analysis to assess, at the same time, the effects of lifestyle interventions on FPG, 2-hour plasma glucose, and diabetes risk in patients with IGT or prediabetes using all available evidence from published RCTs.

## MATERIALS AND METHODS

### Data sources, search strategy, and selection criteria

This study was performed and reported according to the Preferred Reporting Items for Systematic Reviews and Meta-Analysis Statement ( 15 ). RCTs that investigated the effects of lifestyle interventions on FPG, 2-hour plasma glucose, and diabetes risk in IGT or prediabetes patients were eligible for inclusion in this study. An electronic search of PubMed, Embase, and the Cochrane Central Register of Controlled Trials databases was conducted from their inception up to January 2020, using the following core terms: (“prediabetes” OR “impaired glucose tolerance”) AND (“lifestyle intervention”). Ingoing trials were searched on the website http://clinicaltrials.gov/ (US NIH) and the metaRegister of Controlled Trials to identify trials that have been completed but not published. The reference lists from all potentially relevant studies were also manually searched to identify any new eligible study.

All retrieved studies were independently reviewed for eligibility by two authors, and conflicts were resolved by mutual consensus. Studies that met the following criteria were included: 1) Participants: adults with IGT or prediabetes; 2) Intervention: lifestyle interventions focused on diet, behavior, physical activity, or combined; 3) Control: usual care; 4) Outcomes: FPG, 2-hour plasma glucose, and diabetes incidence; and 5) Study design: RCT design. Studies with an observational design were excluded to avoid overestimating the effect estimates of lifestyle interventions.

### Data collection and quality assessment

The information and quality of the included studies were evaluated by two authors, and any disagreement was settled by an additional author after reviewing the original article. The information extracted from the included studies included the first authors’ surname, publication year, country, sample size, mean age, percentage male, mean body mass index (BMI), the status of participants, intervention, control, follow-up duration, and reported outcomes. The quality of the included studies was assessed using the Jadad scale, which is based on randomization, blinding, allocation concealment, withdrawals and dropouts, and use of intention-to-treat analysis ( 16 ). In this study, any study with a score of 4 or 5 was considered as high quality.

### Statistical analysis

The effects of lifestyle interventions on FPG and 2-hour plasma glucose were considered as continuous data, while the risk of diabetes was considered as categorical data. The weighted mean difference (WMD) and relative risk (RR) with corresponding 95% confidence interval (CI) were calculated for the continuous and categorical data, respectively. All pooled results were calculated using the random-effects model to address the underlying variations across the included trials ( 17 , 18 ). Heterogeneity across the included studies was assessed using the *I^2^* and Q statistics, and *I^2^* > 50.0% or *p* < 0.10 was indicative of significant heterogeneity ( 19 , 20 ). The robustness of the pooled conclusions was assessed by a sensitivity analysis ( 21 ). Subgroup analyses for FPG and 2-hour plasma glucose were conducted according to the country, mean age, percentage male, mean BMI, intervention, and study quality, then the differences between subgroups were assessed using interaction *p* -test ( 22 ). Publication biases for the investigated outcomes were assessed by both qualitative (funnel plot) and quantitative methods (Egger and Begg tests) ( 23 , 24 ). The α level for the pooled results was two-sided, and a *p* -value < 0.05 indicated the presence of significant differences between lifestyle interventions and control. STATA/IC 10.0 (StataCorp LLC, Texas, USA) was used to conduct all analyses in this meta-analysis.

## RESULTS

### Literature search

The flowchart of the literature search process for retrieving the relevant studies is displayed in Figure S7 . A total of 1842 potentially relevant articles were identified and screened from the initial electronic searches, of which 1788 articles were excluded because of duplicate titles and irrelevant topics. Fifty-four studies were retrieved for further detailed evaluations, of which 13 RCTs with 3376 individuals with IGT or prediabetes fulfilled the eligibility criteria and were selected for the final meta-analysis ( 25 – 37 ). No additional new eligible study was identified by a manual search of reference lists.

### Study characteristics

The characteristics of the included studies and patients are summarized in Table 1 . The included studies were published between 1997 and 2019, and 69-709 patients were included in each trial. Ten RCTs included patients with IGT, and the remaining three trials included patients with prediabetes. Eight trials were conducted in Europe, four in Asia, and one in the USA. The mean BMI of the included studies ranged from 24.6 to 35.5 kg/m^2^, while the follow-up duration ranged from 12.0 to 72.0 months. The Jadad scale was applied to assess the study quality, one trial had a score of 5, six trials had a score of 4, three trials had a score of 3, and the remaining three trials had a score of 2.

**Table 1:** Characteristics of the included studies and participants

Study	Country	Sample size	Age (years)	Male (%)	BMI (kg/m^2^)	Status of participants	Intervention	Control	Follow-up duration	Study quality
Pan 1997 ( 25 )	China	530	45.0	283 (53.4%)	25.8	IGT	Dietary and/or exercise individual advice	Information about diabetes and IGT	72.0 months	3
Lindahl 1999 ( 26 )	Sweden	186	55.5	69 (37.1%)	30.6	IGT and obesity	Dietary and physical activity intervention	Usual advice	12.0 months	4
Lindstrom 2003 ( 27 )	Finland	522	55.0	172 (33.0%)	31.2	IGT and overweight	Dietary and exercise individual advice	Information about diet and exercise	38.0 months	5
Oldroyd 2006 ( 28 )	UK	69	57.9	39 (56.5%)	NA	IGT	Dietary and physical activity advice	No dietary or physical activity advice	24.0 months	3
Kawahara 2008 ( 29 )	Japan	426	51.4	199 (46.7%)	24.6	IGT	Diabetes education and support	Information about diabetes and IGT	37.0 months	2
Roumen 2008 ( 30 )	Netherlands	106	56.3	58 (54.7%)	29.4	IGT	Dietary and physical activity advice	Information about diet and exercise	36.0 months	4
Yates 2009 ( 31 )	UK	87	65.0	57 (65.5%)	29.2	IGT	Structured education program	Advice leaflet	12.0 months	4
Bhopal 2014 ( 32 )	UK	171	52.5	78 (45.5%)	30.5	IGT	Dietary and physical activity advice	Standardized written and verbal advice	36.0 months	4
O’Dea 2015 ( 33 )	Ireland	50	NA	0 (0.0%)	35.5	IGT	Multidisciplinary team of nurses, dieticians, and physical activity specialists	Standard healthcare advice	12.0 months	2
Van Name 2016 ( 34 )	USA	122	43.4	0 (0.0%)	35.3	Prediabetes	Dietary, behavior, physical activity, and weight loss advice	Usual care	12.0 months	3
Nanditha 2016 ( 35 )	India	709	46.0	NA	25.9	IGT	Dietary and physical activity advice	Standard lifestyle modification advice	24.0 months	4
Gokulakrishnan 2017 ( 36 )	India	150	44.5	91 (60.6%)	28.0	Prediabetes and overweight/obese	Dietary and physical activity advice	Standard lifestyle advice	12.0 months	2
Salas-Salvado 2019 ( 37 )	Spain	248	65.0	NA	32.5	Prediabetes and overweight/obese	Dietary, behavior, physical activity advice	Educational sessions on an ad libitum med-diet	12.0 months	4

### Fasting plasma glucose

**Figure 1:**
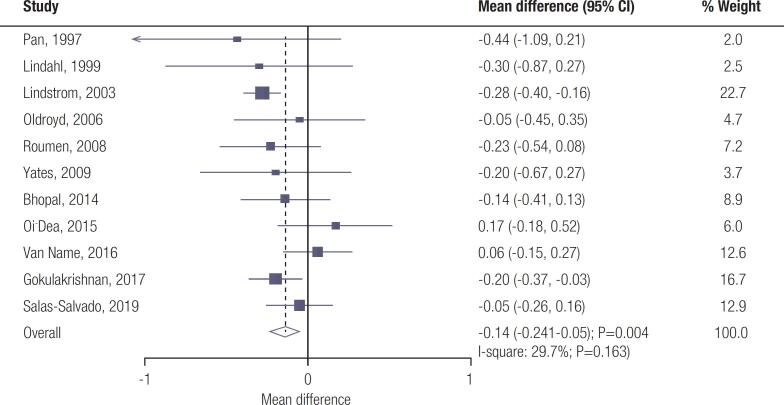
Effect of lifestyle interventions on fasting plasma glucose.

**Figure S1:**
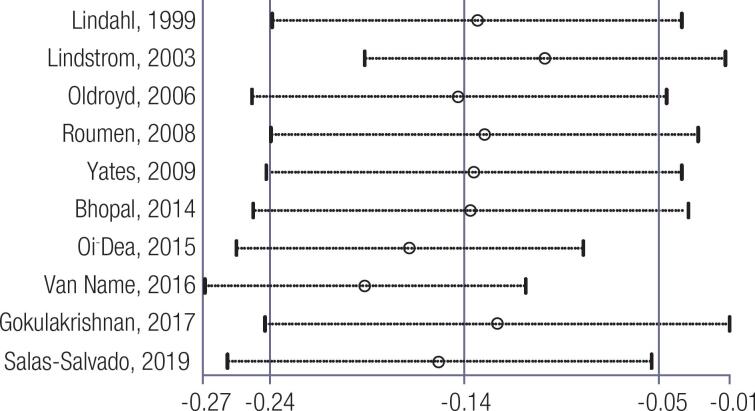
Sensitivity analysis for fasting plasma glucose.

**Figure S2 f5:**
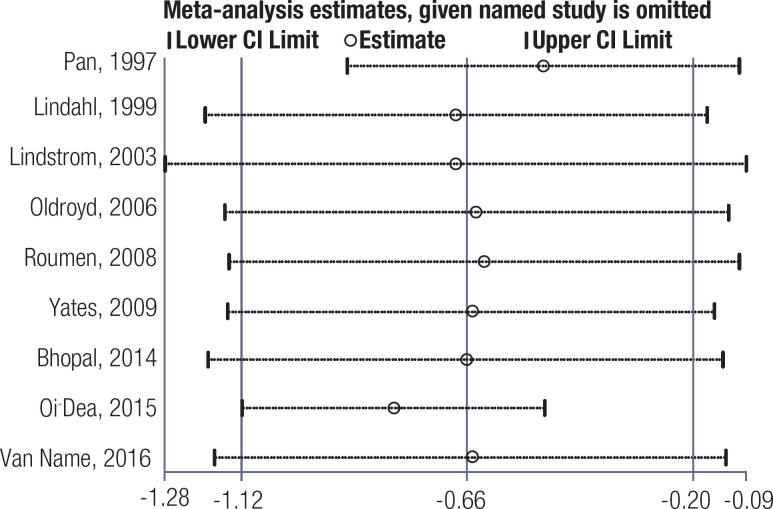
Sensitivity analysis for 2-hour plasma glucose.

**Figure S3:**
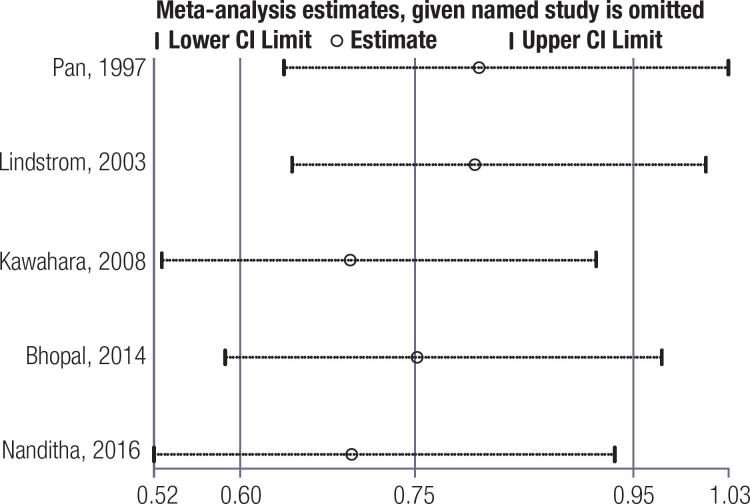
Sensitivity analysis for diabetes.

**Figure S4:**
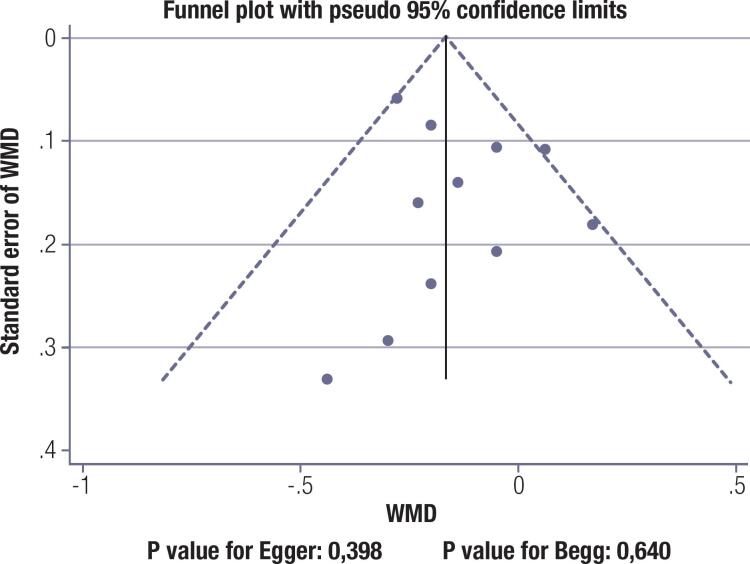
Publication bias for fasting plasma glucose.

**Figure S5 f8:**
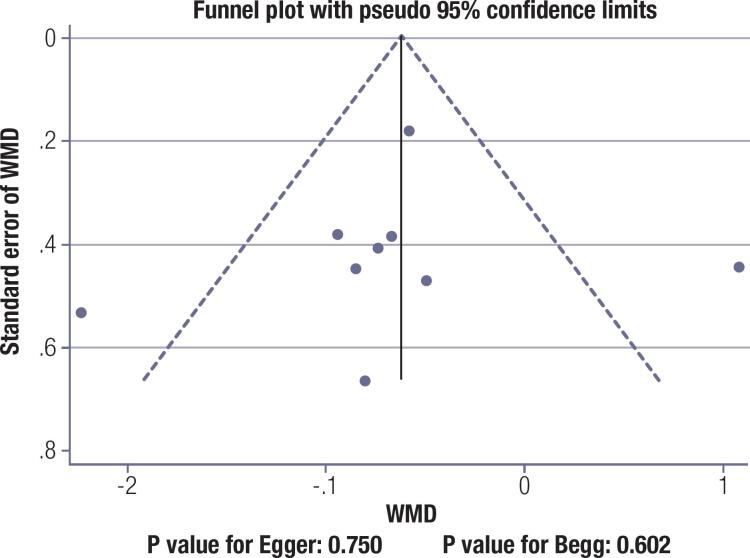
Publication bias for 2-hour plasma glucose.

**Figure S6:**
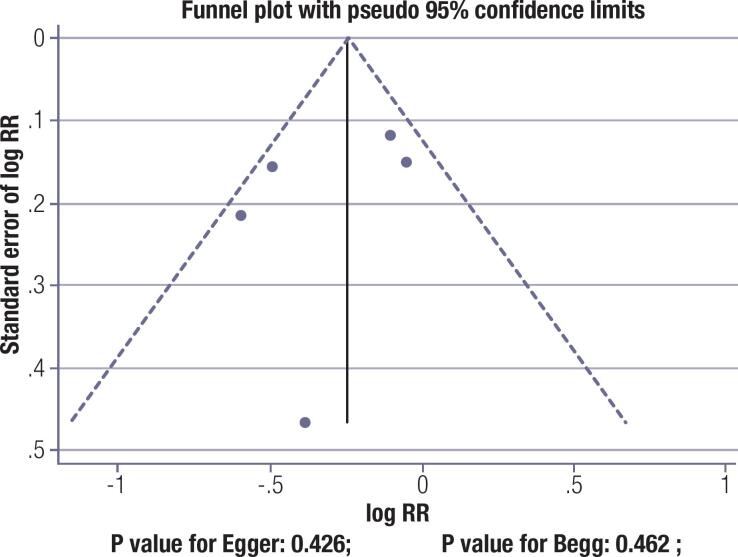
Publication bias for diabetes.

**Figure S7:**
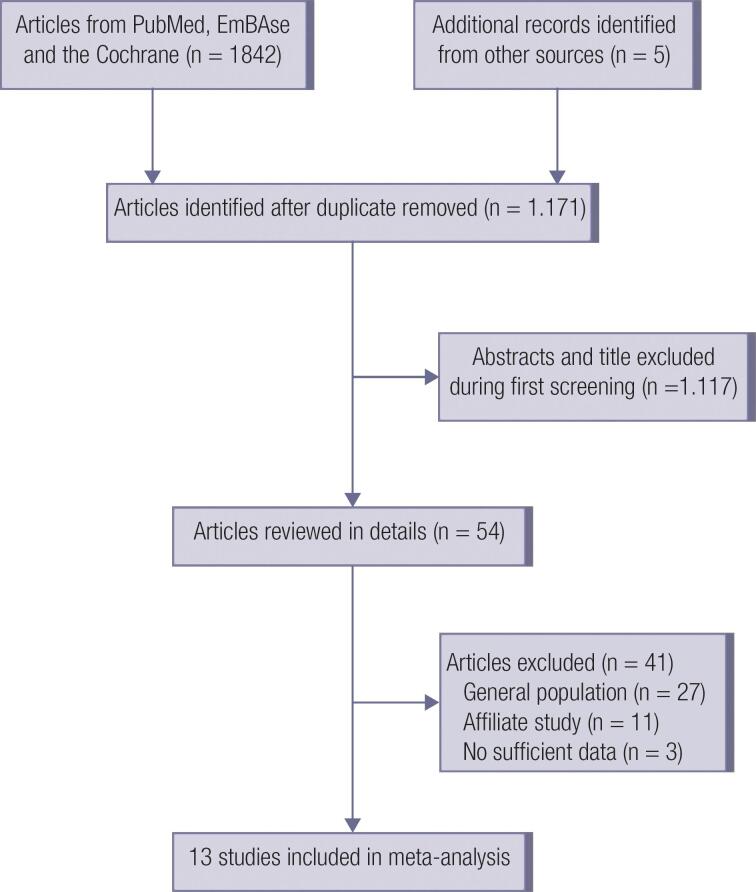
Flow chart of the study selection process.

Data for the effect of lifestyle interventions on FPG levels were available from 11 trials. Lifestyle interventions significantly reduced FPG compared with usual care (WMD: -0.14; 95% CI: -0.24 to -0.05 mmol/L; *p* =0.004; Figure 1 ); insignificant heterogeneity was seen across the included trials ( *I* ^2^ =29.7%; *p* =0.163). The pooled conclusion was robust and not altered by sequentially excluding individual trials ( Figure S1 – S3 ). The subgroup analysis showed that lifestyle interventions were associated with lower FPG for pooled trials conducted in Eastern countries, mean age ≥ 55.0 years, percentage male ≥ 50.0%, mean BMI < 30.0 kg/^2^, the lifestyle interventions comprised of diet and exercise, and study with high quality ( Table 2 ). No significant publication bias for FPG was detected ( *p* -value for Egger: 0.398; *p* -value for Begg: 0.640; Figure S4 – S6 ).

**Table 2:** Subgroup analyses for fasting blood glucose and 2-hour blood glucose

Outcomes	Factors	Subgroup	WMD and 95%CI	p-value	Heterogeneity (%)	p-value for heterogeneity	p-value between subgroups
Fasting blood glucose	Country	Eastern	-0.21 (-0.38 to -0.05)	0.009	0.0	0.482	0.512
		Western	-0.12 (-0.23 to 0.00)	0.054	39.9	0.102	
	Mean age (years)	≥ 55.0	-0.21 (-0.30 to -0.12)	< 0.001	0.0	0.597	0.079
		< 55.0	-0.12 (-0.35 to 0.11)	0.317	56.2	0.102	
	Percentage male (%)	≥ 50.0	-0.20 (-0.33 to -0.07)	0.003	0.0	0.896	0.484
		< 50.0	-0.10 (-0.29 to 0.10)	0.327	65.8	0.020	
	Mean BMI (kg/^2^)	≥ 30.0	-0.09 (-0.25 to 0.07)	0.255	60.9	0.025	0.627
		< 30.0	-0.22 (-0.35 to -0.08)	0.002	0.0	0.917	
	Intervention	Combined	-0.14 (-0.24 to -0.03)	0.009	36.7	0.115	0.229
		Diet	-0.65 (-1.54 to 0.24)	0.152	−	−	
		Exercise	-0.45 (-1.00 to 0.09)	0.104	54.5	0.138	
	Study quality	High	-0.22 (-0.31 to -0.13)	< 0.001	0.0	0.555	0.062
		Low	-0.06 (-0.23 to 0.11)	0.501	41.1	0.147	
2-hour blood glucose	Country	Eastern	-2.23 (-3.27 to -1.19)	< 0.001	−	−	0.002
		Western	-0.51 (-0.91 to -0.11)	0.013	54.5	0.031	
	Mean age (years)	≥ 55.0	-0.66 (-0.93 to -0.40)	< 0.001	0.0	0.957	< 0.001
		< 55.0	-1.45 (-2.90 to 0.01)	0.052	79.8	0.026	
	Percentage male (%)	≥ 50.0	-1.18 (-1.81 to -0.56)	< 0.001	41.7	0.161	0.010
		< 50.0	-0.32 (-0.89 to 0.25)	0.270	69.6	0.011	
	Mean BMI (kg/^2^)	≥ 30.0	-0.32 (-0.89 to 0.25)	0.270	69.6	0.011	0.026
		< 30.0	-1.32 (-2.20 to -0.45)	0.003	55.6	0.105	
	Intervention	Combined	-0.65 (-1.15 to -0.16)	0.010	71.9	0.001	< 0.001
		Diet	-2.48 (-3.58 to -1.38)	< 0.001	−	−	
		Exercise	-1.70 (-3.34 to -0.06)	0.043	75.9	0.042	
	Study quality	High	-0.64 (-0.92 to -0.37)	< 0.001	0.0	0.928	0.772
		Low	-0.67 (-1.93 to 0.60)	0.301	87.5	< 0.001	

### 2-hour plasma glucose

**Figure 2:**
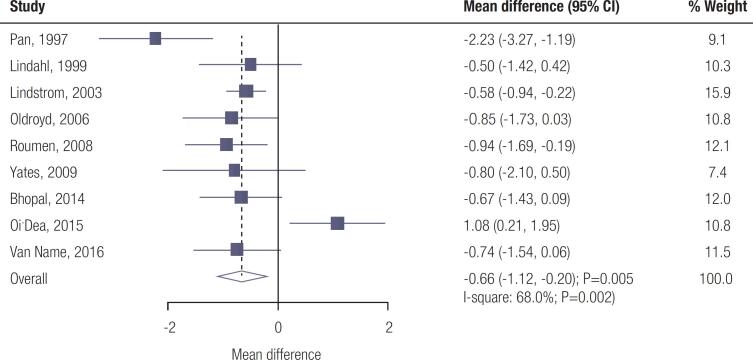
Effect of lifestyle interventions on 2-hour plasma glucose.

Data for the effect of lifestyle interventions on 2-hour plasma glucose levels were available from nine trials. The pooled results showed that lifestyle interventions were associated with lower 2-hour plasma glucose level (WMD: -0.66; 95% CI: -1.12 to -0.20 mmol/L; *p* =0.005; Figure 2 ); significant heterogeneity was detected among the included trials ( *I* ^2^ =68.0%; *p* =0.002). Sensitivity analysis indicated that the conclusion was not altered by sequentially excluding individual trials ( Figure S1 – S3 ). Although significant differences between lifestyle interventions and control on 2-hour plasma glucose level were observed in most subgroups, no significant differences were observed between groups for 2-hour plasma glucose if mean age < 55.0 years, percentage male < 50.0%, mean BMI ≥ 30.0 kg/^2^, and trials with low quality ( Table 2 ). Moreover, the differences between subgroups were statistically significant when stratified by country, mean age, percentage male, mean BMI, and intervention. There was no significant publication bias for 2-hour plasma glucose ( *p* -value for Egger: 0.750; *p* -value for Begg: 0.602; Figure S4 – S6 ).

### Diabetes risk

**Figure 3:**
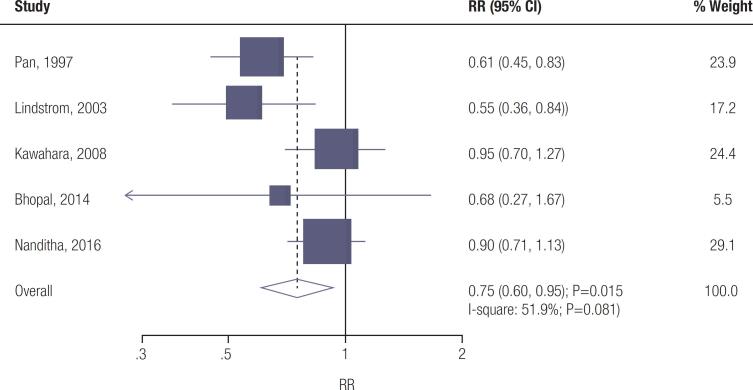
Effect of lifestyle interventions on the risk of diabetes.

Data for the effect of lifestyle interventions on the risk of diabetes were available from five trials. Lifestyle interventions were associated with a reduced risk of diabetes (RR: 0.75; 95% CI: 0.60-0.95; *p* =0.015; Figure 3 ), and significant heterogeneity was detected among the included trials ( *I* ^2^ =51.9%; *p* =0.081). The conclusion for the risk of diabetes was unstable by sequentially excluding individual trials ( Figure S1 – S3 ). No significant publication bias for diabetes was detected ( *p* -value for Egger: 0.426; *p* -value for Begg: 0.462; Figure S4 – S6 ).

## DISCUSSION

The effects of lifestyle interventions on glucose regulation in the general population have been noted in a previous study ( 11 ). Nevertheless, whether lifestyle interventions can influence both FPG and 2-hour plasma glucose in patients with IGT or prediabetes remained poorly known, especially with respect to different regions, sex, and BMI. In this study, the effects of lifestyle interventions on FPG, 2-hour plasma glucose, and diabetes risk in IGT or prediabetes patients were investigated. A total of 3376 individuals with IGT or prediabetes from 13 RCTs were identified, and the pooled results suggested that lifestyle interventions significantly improved FPG and 2-hour plasma glucose and prevented the risk of diabetes. Moreover, the effects of lifestyle interventions in adults with IGT or prediabetes were influenced by country, mean age, percentage male, mean BMI, intervention, and study quality. Taken together, the results indicate that non-pharmacological lifestyle changes are sufficient to induce changes in glucose metabolism and prevent the development of T2DM in many individuals. Such interventions are cost-effective, both for the patients and the healthcare systems, and the costs associated with patient education in T2DM prevention through lifestyle changes are lower than the costs associated with T2DM care ( 38 , 39 ). Therefore, such prevention programs play an important role in public health.

The Diabetes Prevention Program (DPP) previously showed that lifestyle changes could decrease the incidence rate of diabetes in a very successful manner ( 8 – 10 ). A previous systematic review and meta-analysis conducted by Gong and cols. ( 14 ) included nine RCTs and suggested that lifestyle interventions based on diet, physical activity, behavior, or combinations could improve FPG and 2-hour plasma glucose. The significant effects of lifestyle interventions on FPG mainly focused on physical activity or combining diet with physical activity, and the significant effects on 2-hour plasma glucose mainly focused on physical activity or diet. Still, several limitations of that study should be mentioned: 1) the analysis applied standardized mean difference as an effect estimate, and the exact difference between lifestyle interventions and control was not obtained; 2) the pooled analyses included several studies that reported the same population, so the results of the meta-analysis might be overestimated; and 3) the effects of lifestyle interventions on FPG, and 2-hour plasma glucose based on individuals’ characteristics were not illustrated. Therefore, the current meta-analysis was conducted to assess the potential role of lifestyle interventions on glucose regulation and diabetes in adults with IGT or prediabetes.

The pooled results showed that lifestyle interventions were associated with lower FPG and 2-hour plasma glucose. Only a few included studies reported similar results on the levels of FPG and 2-hour plasma glucose. Two trials found that dietary and exercise advice was associated with lower 2-hour plasma glucose, while no significant difference was found between the groups on FPG ( 25 , 30 ). A study conducted by Lindstrom and cols. found that combined dietary and exercise individual advice was associated with lower levels of FPG and 2-hour plasma glucose ( 27 ). Gokulakrishnan and cols. found that dietary and exercise advice was associated with lower FPG but had no significant effect on 2-hour plasma glucose ( 36 ). These discrepancies could be explained by the intensity of interventions, the duration of interventions, and follow-up duration. Nevertheless, FPG and 2-hour plasma glucose are simple screening tests for T2DM and are available at all hospitals ( 40 , 41 ). These tests should be performed routinely in patients with IGT for the early detection of a progression to T2DM.

The summary results of this study found that dietary and/or exercise individual advice were associated with a reduced risk of diabetes, and two of the included studies reported similar results ( 25 , 27 ). The effect of lifestyle interventions on the risk of diabetes was significantly correlated with multiple lifestyle changes, and the subjects who managed to reach most lifestyle targets could achieve more evidence effect. Moreover, the interventions of diet and exercise could affect insulin resistance by increasing insulin-mediated glucose disposal in muscles and weight loss ( 42 , 43 ). However, this conclusion was unstable, and the potential reason for this could be that this result was reported from only five included trials.

The subgroup analyses showed that the effects of lifestyle interventions on glucose regulation might differ when stratified by country, mean age, percentage male, mean BMI, intervention, and study quality. The effects of lifestyle interventions were more evident in trials conducted in Eastern countries, elderly patients, male patients, combined diet and exercise, lower BMI, and high quality of the study. Several factors could explain these results: 1) dietary differences exist between Eastern and Western countries, and the lifestyle targets could influence the effects of lifestyle interventions; 2) the adherence of lifestyle interventions could be affected by age and the percentage of male patients; 3) the changes of lifestyles may be related to the content of interventions; 4) the BMI is significantly correlated with the risk of diabetes, and these results suggested that lifestyle interventions should be applied for patients with lower BMI; and 5) the study quality is significantly correlated with the reliability of pooled conclusions.

The advantages of this study should be highlighted. First, the analysis was based on RCTs, and the evidence level of pooled results was the highest. Second, the results of this study were quantitatively determined by a large sample size, which was more robust than any individual trial. Third, the effects of lifestyle interventions on FPG and 2-hour plasma glucose stratified by country, mean age, percentage male, mean BMI, intervention, and study quality were conducted to achieve comprehensive results of lifestyle interventions in subpopulations. This study also had several limitations: 1) the heterogeneity for 2-hour plasma glucose was not fully explained by the sensitivity and subgroup analyses; 2) only a few included trials reported the incidence of diabetes, and the results should be confirmed by additional RCTs; 3) the analysis was based on pooled data, and individual data was not available; 4) this study was based on published studies, and publication bias was inevitable; and 5) because of the strict eligibility criteria, some studies, including those about the DPP ( 8 – 10 ), for example, could not be included.

In conclusion, this study reinforces that lifestyle interventions could significantly improve FPG and 2-hour plasma glucose and reduce the risk of diabetes in adults with IGT or prediabetes. Further large-scale RCTs should be conducted to assess the long-term effects of lifestyle interventions on the risk of diabetic complications in adults with IGT or prediabetes.

## Supplemental materials

[Fig f5]

[Fig f8]
